# ATP Hydrolysis Induced Conformational Changes in the Vitamin B_12_ Transporter BtuCD Revealed by MD Simulations

**DOI:** 10.1371/journal.pone.0166980

**Published:** 2016-11-21

**Authors:** Chao Pan, Jingwei Weng, Wenning Wang

**Affiliations:** Shanghai Key Laboratory of Molecular Catalysis and Innovative Materials, Department of Chemistry, and Institutes of Biomedical Sciences, Fudan University, Shanghai, P. R. China; Massachusetts Institute of Technology, UNITED STATES

## Abstract

ATP binding cassette (ABC) transporters utilize the energy of ATP hydrolysis to uni-directionally transport substrates across cell membrane. ATP hydrolysis occurs at the nucleotide-binding domain (NBD) dimer interface of ABC transporters, whereas substrate translocation takes place at the translocation pathway between the transmembrane domains (TMDs), which is more than 30 angstroms away from the NBD dimer interface. This raises the question of how the hydrolysis energy released at NBDs is “transmitted” to trigger the conformational changes at TMDs. Using molecular dynamics (MD) simulations, we studied the post-hydrolysis state of the vitamin B_12_ importer BtuCD. Totally 3-μs MD trajectories demonstrate a predominantly asymmetric arrangement of the NBD dimer interface, with the ADP-bound site disrupted and the ATP-bound site preserved in most of the trajectories. TMDs response to ATP hydrolysis by separation of the L-loops and opening of the cytoplasmic gate II, indicating that hydrolysis of one ATP could facilitate substrate translocation by opening the cytoplasmic end of translocation pathway. It was also found that motions of the L-loops and the cytoplasmic gate II are coupled with each other through a contiguous interaction network involving a conserved Asn83 on the extended stretch preceding TM3 helix plus the cytoplasmic end of TM2/6/7 helix bundle. These findings entail a TMD-NBD communication mechanism for type II ABC importers.

## Introduction

ABC transporters constitute one of the largest protein superfamilies in all organisms [1]. They share a common building block, namely the ATP-binding cassette that binds and hydrolyzes ATP. The released energy is utilized to uni-directionally transport substrate across cell membranes. ABC importers are often involved in nutrient uptake, pathogenicity and virulence [2], whereas ABC exporters are associated with lipid transport, multidrug resistance and genetic diseases [3–5]. ABC transporters minimally consist of four domains, including two transmembrane domains (TMDs) and two ATP-binding cassettes (also called nucleotide-binding domains, NBDs). TMDs traverse cell membrane and enclose a substrate translocation pathway at their interface. The translocation pathway could adopt at least two major conformations: the inward-facing one toward the cytoplasm and the outward-facing one toward the periplasm or the extracellular side. The NBDs are immersed in the cytosol, bind and hydrolyze ATP to trigger the conformational changes of the translocation pathway in TMDs [6].

BtuCD is the most extensively studied type II ABC importer that mediates the uptake of vitamin B_12_ (abbreviated as B_12_ hereafter) across the inner membrane in *E*. *coli*. Besides the four basic domains, a cognate periplasmic binding protein, BtuF [7, 8], is also required to maximize transport rate [9] as is found in many ABC importers. BtuCD has been subjected to many biochemical and structural characterizations [9–19]. Four different conformational states of BtuCD and a fifth conformational state of MolBC (also called HI1470/1, a homogenous molybdate/tungstate importer from *Haemophilus influenza*) have been identified from crystal structures, based on which a mechanism of B_12_ transport cycle in BtuCD was proposed [18]. A productive transport cycle starts with ATP-bound BtuCD whose translocation pathway adopts an outward-facing conformation ready to accept B_12_ [18]. Subsequent association of ligand-bound BtuF triggers the release of B_12_ to BtuCD and closure of the periplasmic gate, leading to the occluded state (also called the occ-BtuCD state) [17]. Then ATP hydrolysis results in conformational transition toward the inward-facing conformation similar with that in the crystal structure of MolBC [18], which would facilitate the release of substrate into cytoplasm [20]. Afterwards, the transporter undergoes conformational transition to the asymmetric conformational state with both sides of the translocation pathway occluded [15]. The subsequent dissociation of BtuF is followed by reopening of the periplasmic gate [19], preparing for binding new ATPs and entering the next round of transport.

**Fig 1:**
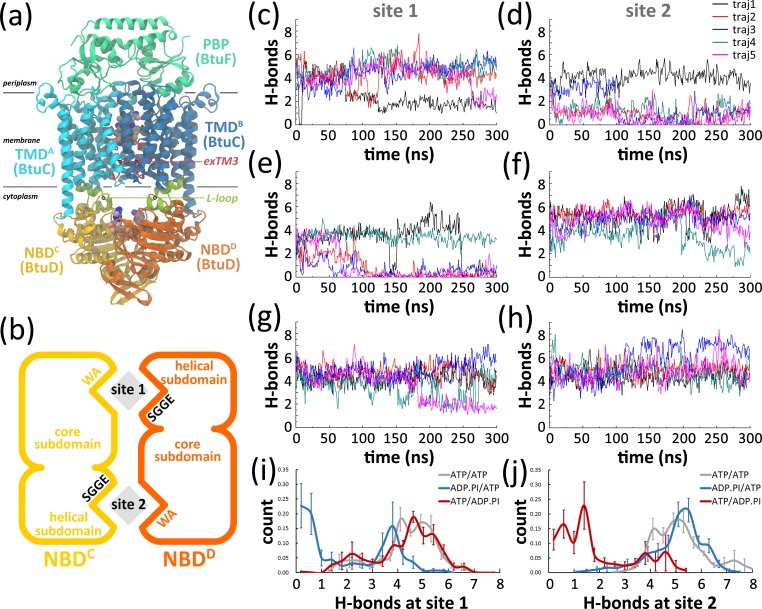
(a) Front view of BtuCD-F complex in the occluded state. Vitamin B_12_, Mg^2+^, inorganic phosphate and nucleotides are represented by van der Waals model. (b) Schematic view of the NBD dimer and the nucleotide binding sites. The nucleotides are represented by gray diamonds. (c-d) Variations of the number of hydrogen bonds (H-bonds) formed between nucleotide and the corresponding signature motif at two active sites in the **ATP/ADP.IP** trajectories along simulation time. (e-f) Variations of the number of H-bonds at two active sites in the **ADP.IP/ATP** trajectories. (g-h) Variations of number of H-bonds at two active sites in the **ATP/ATP** trajectories obtained from our previous work [23]. (i-j) The distributions of H-bond numbers at the two active sites in three systems. H-bond is defined as the donor and acceptor atoms are within 3.5 Å from each other, and the angle formed by the donor, hydrogen, and acceptor atoms is less than 60° from 180°. The plotted number of H-bond is averaged over 10 consecutive snapshots.

Among these conformations, the occ-BtuCD state is regarded as a crucial step of the transport cycle, in which the transporter simultaneously loads the shipment B_12_ and the energy source ATPs (Fig 1A). Two ATPs are bound at the nucleotide-binding sites (also called active sites) at the fully closed NBD dimer interface [2, 17, 21]. The NBD dimer interface follows a consensus binding mode for nucleotides across ABC superfamilies. The Rec-A core subdomain of one NBD participates by conserved motifs, such as the Walker A (WA, also called P-loop) motif which wraps the phosphate moieties through extensive hydrogen bond interactions, and the A-loop which establishes π-π stacking with the adenosine moiety [22]. The signature motif on the helical subdomain of the other NBD constitutes the other half of active site by stretching across the NBD interface and binding to the adenine, ribose and γ-P moiety of nucleotide. The TMDs of BtuCD are characterized by 10 transmembrane (TM) helices in each domain, common to all type II ABC importers. The middle part of the translocation pathway at TMD dimer interface is capable of accommodating B_12_ in the occ-BtuCD state [17, 23], and the accesses to periplasm and cytoplasm are occluded by the periplasmic gate and cytoplasmic II gate, respectively. The periplasmic gate is composed of TM5a helices and the C-terminal ends of TM5 helices, while the cytoplasmic gate II is mainly constituted by the extended stretches preceding TM3 helix (exTM3s).

The transition from the occ-BtuCD state to the inward-facing state after ATP hydrolysis raises questions on how ATP hydrolysis drives the separation of NBD dimer interface and how it triggers the conformational changes in the translocation pathway which is more than 30 angstroms away. It was noticed that a cytoplasmic loop between TM6 and TM7 helices that folds into two short helices (L1 and L2) shares extensive contacts with the complementary groove on NBD surface (Fig 1A). The loop was named as “L-loop” and proposed to be critical in TMD-NBD communication [19]. However, the molecular mechanism of the allosteric communication is still largely unknown. Moreover, whether the MolBC structure reflects a physiologically relevant state is still questionable, because absence of nucleotide at active sites would be very rare under physiological condition given that ATP concentration in cell far exceeds the binding affinity of ABC transporters for ATP [24–26].

Molecular dynamics (MD) simulations have been used to study the post-hydrolysis state of full-length ABC transporters [27–31] and isolated NBDs [32–39]. For BtuCD, most works focused on the outward-facing state [40–44]. In this work, we generated the post-hydrolysis state of BtuCD-F complex with one of its active sites binding ATP and the other binding ADP plus inorganic phosphate (IP). Totally 3-μs trajectories were accumulated to monitor the conformational changes of the state. Most trajectories demonstrate an asymmetric arrangement of NBD interface with only the ADP-bound site disrupted. Changes in TMDs, including separation of the L-loops and opening of the cytoplasmic gate II were also observed. A contiguous interaction network was identified at the cytoplasmic side of TMD, which couples the motions of L-loops and cytoplasmic gate II.

## Methods

### System Setup

The crystal structure of the occluded state (PDBID: 4FI3) was used as the initial structure of BtuCD-F complex. The mutated residues were restored to their native types and the ATP mimics (AMP-PNP) were replaced by ATP and ADP.IP. We suppose that either ATP may be hydrolyzed, leading to the **ATP/ADP.IP** or **ADP.IP/ATP** system. The vitamin B_12_ was placed inside the translocation cavity as described in our previous work [23]. The complex was first inserted into a preequilibrated palmitoyl-oleoyl phosphatidyl-ethanolamine (POPE) bilayer with 340 lipids by the shrinking method [45], and then placed in a rectangular box with the size of 107×106×168 Å^3^, solvated and ionized by 0.1 mol/L NaCl. The post-hydrolysis system contains totally 194,137 atoms, including 323 POPE lipids, 1 ATP, 1 ADP, 1 IP, 2 Mg^2+^, 43,888 water molecules, 117 sodium ions and 136 chloride ions. The system was then energy-minimized by 10000 steps, equilibrated for 40 ps with all the protein, ligand and lipid atoms fixed, and further equilibrated for 2.1 ns with only the heavy atoms of proteins and ligands harmonically restrained. The final structure of the position-restraining simulation was used as the initial structure for the production runs.

### Simulation details

The MD simulations were performed with Gromacs 4.5 [46]. Proteins were described by amber99sb force field [47], lipids by Slipid force field [48, 49], and water molecules by TIP3P model [50]. The parameters of B_12_ were the same as used in our previous work [23]. The parameters of IP were obtained from the work of Steinbrecher *et al*. [51]. Temperature was maintained at 310 K by the weak coupling method with a time constant of 0.1 ps, and pressure was maintained at 1 bar with a time constant of 5 ps [52]. Semi-isotropic pressure coupling was adopted. All bonds were constrained, allowing a time step of 2 fs. Electrostatic interactions were evaluated using the particle mesh Ewald method [53]. The cutoff of VDW interactions was set to 12 Å. Five 300-ns trajectories were produced for each of the two hydrolysis systems (i.e. the **ATP/ADP.IP** and the **ADP.IP/ATP** system). The coordinates of proteins and ligands were saved every 50 ps. Analysis of trajectories and visualization of molecular structures were done by VMD [54].

## Results

Conventional MD simulations were conducted for two post-hydrolysis systems based on the occluded state of BtuCD-F, supposing one of the binding nucleotides is hydrolyzed to ADP.IP. Hydrolysis may occur at either active sites, leading to the **ATP/ADP.IP** (hydrolysis occurs at site 2) and **ADP.IP/ATP** (hydrolysis occurs at site 1) systems (Fig 1B). Note that these two systems are not symmetrically identical since the two lobes of BtuF are different. Five 300-ns trajectories were produced for each system. The trajectories of the system with ATP bound in both sites (the **ATP/ATP** system) were obtained in our previous work [23] and are used here as control.

### ATP Hydrolysis Leads to Asymmetric opening of the NBD Interface

ATP hydrolysis is supposed to destabilize the fully closed arrangement of the active sites according to previous simulations of ABC transporters [33,34,36]. As nucleotides (ATP or ADP) are always tightly bound to the WA motif in all the trajectories (S1 Table), we counted the number of hydrogen bonds (H-bonds) formed between nucleotide and corresponding signature motif to monitor the state of active sites (Fig 1C–1J). In the **ATP/ADP.IP** system, the number of H-bonds fluctuated around five at the ATP-bound site in most trajectories (Fig 1C), whereas the number for the ADP-bound site decreased to one in four of the trajectories (Fig 1D). The statistics of H-bond number also shows a sharp decrease at the ADP-bound site (Fig 1J). Evident loss of contact between nucleotide and signature motif was also observed at the ADP-bound sites in four trajectories of the **ADP.IP/ATP** systems (Fig 1E), while the interactions at the ATP-bound site were mostly preserved (Fig 1F). The decrease of H-bond number at the ADP-bound site in the **ADP.IP/ATP** can be clearly seen in Fig 1I. In contrast to the post-hydrolysis systems, most H-bonds at both active sites were retained in the **ATP/ATP** systems (Fig 1G–1J), demonstrating the crucial role of ATP in stabilizing the full closed NBD interface.

We also use the distance between the mass center of alpha and beta phosphate groups of nucleotide and the mass center of the corresponding signature motif ^128^SGGE^131^, d_nuc-sig_, to monitor the variations at active sites (S1 Fig). The results demonstrate similar asymmetrical behavior of NBD interface as ADP-bound site was more frequently disrupted relative to the ATP-bound site (S1A–S1D Fig). The most evident asymmetric arrangement was observed in trajectory 5 of the **ADP.IP/ATP** system. The d_nuc-sig_ of the ADP-bound site surpassed 13 Å (S1C Fig), whereas d_nuc-sig_ of the other site remained less than 8 Å (S1D Fig). However, the widely opened state did not last for long time, and oscillated back to partially opening state (d_nuc-sig_ = ~10 Å) after a few nanoseconds. The opening magnitude of the ADP-bound site in our simulations varied from 3 to 6 Å, which is similar to the MD simulation studies of the *Staphylococcus aureus* multidrug exporter Sav1866 [29, 32], the maltose importer MalFGK [30], and its MalK dimer [36], but is less than that of the NBD dimer of MJ0796 from *Methanococcus jannaschii* [33], possibly due to the high temperature environment the protein stays in [55].

**Fig 2:**
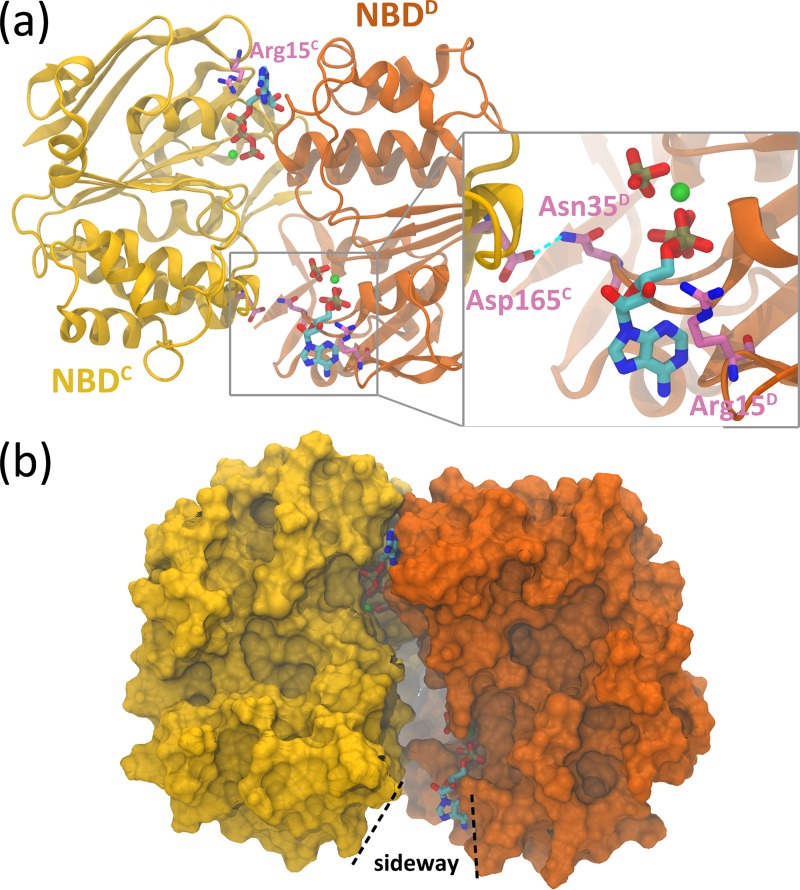
Snapshot at 114.80 ns of the ADP.IP/ATP trajectory 5. (a) The binding nucleotides at the sites. NBDs are represented by ribbons, and TMDs are omitted for clarity. The nucleotide, IP and Mg^2+^ ions are represented by the licorice mode with carbon colored by cyan, oxygen by red, nitrogen by blue, phosphate by tan and magnesium by green. The ADP-bound site is zoomed in. (b) Sideway at the ADP-bound site. NBDs are represented by their solvent accessible surfaces.

For a more detailed view of the changes at the NBD interface, the snapshot of the most opening site of the **ADP.IP/ATP** system is shown in Fig 2. Residues in two NBDs are differentiated by the chain IDs (C or D). At 114.80 ns of the **ADP.IP/ATP** trajectory 5, the ADP-bound site opened most widely with d_nuc-sig_ equaling to 13.5 Å (S1C Fig). All the interactions between ADP and the signature motif were disrupted (Figs 1E and 2A), and the nucleotide became more exposed with its solvent accessible surface area rising by ~270 Å^2^. A sideway could be observed at the NBD interface (Fig 2B), which is large enough for ADP to exit from the site. Moreover, its adenine moiety flipped away from Arg15 on the A-loop and disrupted the interaction with it (Fig 2A). The flipping motion could be regarded as the first step of ADP dissociation and was also observed in the simulations of the ADP/ATP bound MJ0796 dimer [33]. Though ADP itself fully detached from the signature motif upon the opening motion, the inter-domain interactions were still maintained at the opened site, such as the hydrogen bond between the side chain of Asn35^C^ on the WA motif and the carboxyl group of the conserved Asp165^D^ on the juxtaposing D-loop (Fig 2A). A similar inter-domain WA-Q-loop hydrogen bond interaction was also observed at the ADP-bound site in MalK dimer [36]. The preserved inter-domain interaction could be utilized to retain the relative position of the WA and the signature motifs, and may be responsible for the fast oscillation motion of the ADP-bound site during the simulations (S1C Fig).

Throughout the simulations, Mg^2+^ ion kept interacting with the β- and/or γ-phosphate groups of nucleotide or the IP, as the cation was always within 3.5 Å from the phosphorus atoms (S2 Fig). The proximity of IP to Mg^2+^ ion also indicates that IP did not exit from ADP-bound site during our simulations (also demonstrated by Fig 2A). Retention of Mg^2+^ and IP at the active site was also observed in the simulations of the post-hydrolysis states of MalK dimer [36] and the NBD dimer of MJ0796 [34]. It should be noted that although the hydrolysis products will ultimately leave the binding site for receiving a new ATP, the process may take quite a long time and the observed retention of Mg^2+^ and IP may be due to the limitation of our simulation as well as other simulation works.

Although the trajectories generally showed asymmetric disruption at the ADP-bound site, exceptions were also observed. For example, in trajectory 4 of the **ADP.IP/ATP** system, the ADP-bound site remained almost intact (Fig 1E), whereas more than half of the H-bonds at the ATP-bound site were lost in the last 70 ns (Fig 1F). Case is similar for **ATP/ADP.IP** trajectory 1, though the detachment at the ATP-bound site initiated earlier (Fig 1C). In the last 45 ns of **ATP/ADP.IP** trajectory 5, both the ATP-bound and ADP-bound sites were partially disrupted (Fig 1C and 1D), indicating a probability of disruption at both sites.

### Conformational Changes of the L-loops and Cytoplasmic Gates II upon ATP Hydrolysis

**Fig 3:**
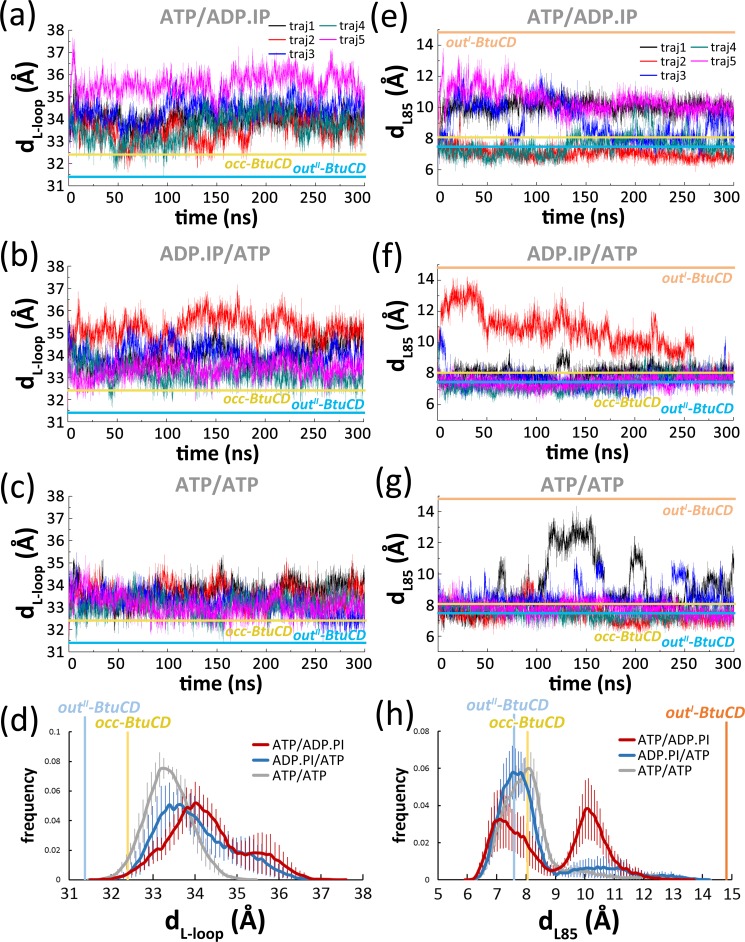
Variations of d_L-loop_ in five 300-ns trajectories of the **ATP/ADP.IP** system (a) the **ADP.IP/ATP** system (b) and the **ATP/ATP** system (c) along simulation time. d_L-loop_ is defined as the distance between the mass centers of two L-loops on each TMD. (d) Distribution of d_L-loop_ in three systems. The error bars are estimated by bootstrap method. Variations of d_L85_ in five 300-ns trajectories of the **ATP/ADP.IP** system (e), the **ADP.IP/ATP** system (f) and the **ATP/ATP** system (g) along simulation time. d_L85_ is defined as the distance between C_α_ atoms of Leu85 on the exTM3 stretches. (h) Distribution of d_L85_ in three systems. Data of the **ATP/ATP** system were obtained from our previous work [23]. The values of the crystal structures of the occluded state (occ-BtuCD, PDBID: 4FI3), the nucleotide-free BtuCD (out^I^-BtuCD, PDBID: 1L7V), and the AMP-PNP-bound BtuCD (out^II^-BtuCD, PDBID: 4R9U) are denoted by horizontal or vertical lines, respectively.

Conformational changes triggered by ATP hydrolysis were also observed in TMDs. One of the notable changes occurs at the L-loop. The distance between the mass centers of two L-loops, d_L-loop_, was longer than 35.5 Å in three trajectories of the **ATP/ADP.IP** system (Fig 3A) and in three trajectories of the **ADP.IP/ATP** system (Fig 3B). For comparison, d_L-loop_ mostly fell within 32 to 35 Å and never exceeded 35.5 Å in the **ATP/ATP** system (Fig 3C and 3D). The distributions of d_L-loop_ demonstrate a clear shift to larger value in the post-hydrolysis systems (Fig 3D). The most evident separations took placed in **ATP/ADP.IP** trajectory 5 and **ADP.IP/ATP** trajectory 2. In both trajectories, d_L-loop_ kept fluctuating around 35.5 Å and the maximum value exceeded 37 Å (Fig 3A and 3B). Significant loss of H-bonds at the ADP-bound site was also observed in these trajectories (Fig 1D and 1E), suggesting that there is a coupling relationship between the motions of the L-loops and the active site opening (see below).

The conformational changes of cytoplasmic gate II were monitored by the distance between C_α_ atoms of Leu85 on exTM3 stretches (d_L85_), the residue that constricts the gate II (Fig 3E–3H) [17]. In the **ATP/ADP.IP** system (Fig 3E), d_L85_ was larger than 10 Å in all five trajectories and the boosting of d_L85_ lasted for more than 280 ns in two of the trajectories. Opening motion with similar magnitude was also observed in two trajectories of the **ATP/ATP** system (Fig 3G), but it was less frequent and lasted for only 65 ns at most. The distribution of d_L85_ distribution in the **ATP/ADP.IP** system exhibit a second peak at longer distance around 10 Å (Fig 3H), further indicating that ATP hydrolysis indeed facilitates gating. Among all the trajectories, **ATP/ADP.IP** trajectory 5 exhibits the most long-lasting opening of gate II, in which d_L85_ increased at the very beginning of simulation and fluctuated around 10 Å till the end (Fig 3E). Notably, **ATP/ADP.IP** trajectory 5 also shows the most widely separated L-loops of the system (Fig 3A), indicating coupling motions of the cytoplasmic gate II and the L-loops.

Continuous opening of cytoplasmic gate II was also observed in the **ADP.IP/ATP** trajectory 2, whose d_L85_ increased to more than 10 Å at the very beginning and reached a maximum of over 14.2 Å at 29.90 ns (Fig 3F). The opening state lasted for more than 250 ns and the gate occluded at 268 ns. The opening also results in some populations of d_L85_ around 10–11 Å, although significantly less than that of the **ATP/ADP.IP** system (Fig 3H). Again, this trajectory has the most separated L-loops of the system (Fig 3B), suggesting the coupling relationship.

Though the opening of cytoplasmic gate II was observed in the post-hydrolysis systems, the cytoplasmic end of translocation pathway is still less opened than that observed in the inward-facing state of *Haemophilus influenzae* molybdate/tungstate transporter MolBC (S3 Fig) [20], and is still impermeable for the large-sized substrate. Consistently, we found that the substrate kept 10~12 Å away from the periplasmic gate (S4 Fig), and no further progression towards the cytoplasm was observed in the two trajectories with the most long-lasting opening cytoplasmic gate II. From these observations, we infer that the substrate translocation is a relatively slow process and would not occur on the sub-microsecond time scale. Longer simulation time may be required to capture the translocation process of the substrate.

### Molecular Mechanism of the Coupling Motions at the TMD-NBD Interface and at the Cytoplasmic End of TMD

From the trajectories of the post-hydrolysis systems, we could find that disruption of the ADP-bound site, evident separation of the L-loops, and long-lasting opening of cytoplasmic gates II would take place in the same trajectory. However, how these motions are coupled with each other is still unclear. Here, we analyze the **ATP/ADP.IP** trajectory 5 and **ADP.IP/ATP** trajectory 2, which show the most evident opening motion of the cytoplasmic gate II, to decipher the molecular details behind these coupling motions.

**Fig 4:**
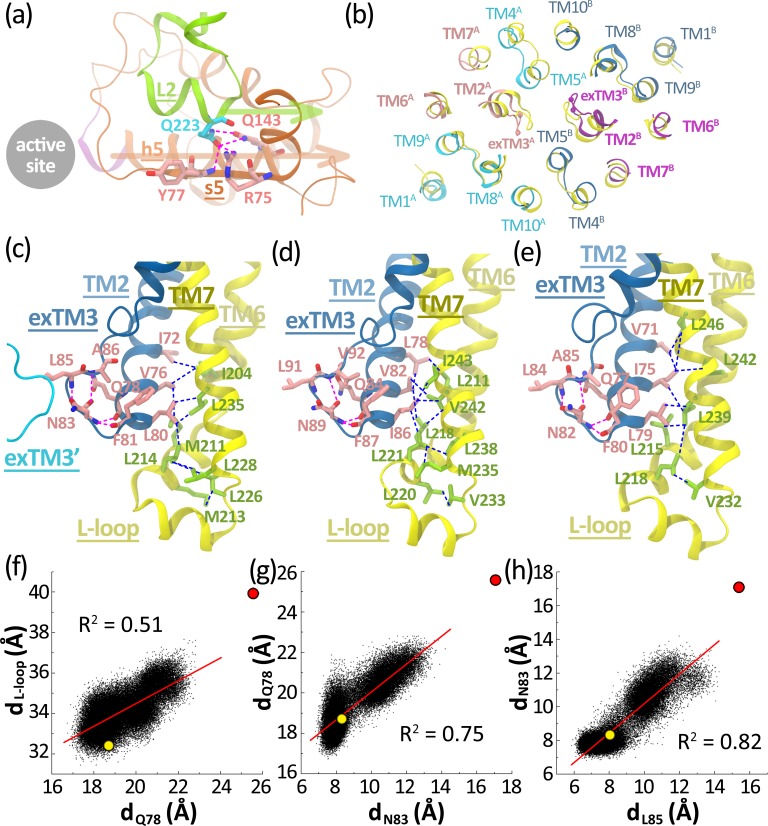
(a) Close view of the TMD-NBD interface in the occluded state of BtuCD. The importer is represented by cartoon mode, and the signature motif (^128^SGGE^131^) is highlighted by purple. The residues participating in the hydrogen bond interactions across the interface are represented by the licorice mode with nitrogen colored by blue and oxygen colored by red. The carbon atoms on the L-loop are colored by cyan and those on the NBD are by pink. The hydrogen bond interactions are marked by magenta dashed lines. The retraction motions of h5 helix and L-loop away from active site are denoted by arrows. (b) Snapshot of the cytoplasmic ends of TMDs at 29.90 ns of **ADP.IP/ATP** trajectory 2 which has the largest d_L85_ (14.2 Å). The importer is represented by ribbons and is viewed from the periplasm. TMDs of the snapshot are colored by cyan and blue, respectively, and TM2, exTM3, TM6 and TM7 on each TMD are highlighted by pink and purple, respectively. Leu85 and Asn83 on exTM3 stretch are represented as balls. TMDs of the crystal structure are colored yellow. The L-loops are omitted for the sake of clarity. (c-e) The contiguous interaction network composed of the Asn83-involved (or equivalent residue-involved) hydrogen bond interactions and the hydrophobic interactions at the cytoplasmic end of TM2/6/7 helix bundle plus the L-loop in the crystal structures of AMP-PNP-bound BtuCD-F (c), the *Haemophilus influenzae* molybdate/tungstate transporter MolBC (d), and the *Yersinia pestis* heme transporter HmuUV (e). The exTM3 stretch and TM2 helix are colored by blue, and the carbon atoms of the residues on them are colored by pink. The L-loop, TM6 and TM7 helices are colored yellow, and the carbon atoms of the residues on them are colored by green. The other helices of TMD are omitted for clarity. The hydrogen bond interactions are marked by magenta dashed lines, and the hydrophobic interactions are marked by light blue dashed lines. (f) Projections of all the trajectories of the post-hydrolysis systems on the 2-dimensional space spanned by d_Q78_ and d_L-loop_. (g) Projections of the trajectories on the 2-dimensional space spanned by d_N83_ and d_Q78_. (h) Projections of all the trajectories of the post-hydrolysis systems on the 2-dimensional space spanned by d_L85_ and d_N83_. The projections of the occ-BtuCD state and the inward-facing state of MolBC (equivalent residues) on the figures are denoted by yellow and red circles, respectively.

We first inspect the TMD-NBD interface as the L-loop participates in inter-domain interactions through a number of hydrogen bonds (S2 Table). The strongest hydrogen bond interactions are formed between Gln223 on the second helix of L-loop (L2) and the residue on the C-terminal end of h5 helix (Gln143) plus those on the s5 strand (Arg75 and Tyr77) (Fig 4A). These interactions are absolutely stable in all trajectories, and are hardly affected by the changes of nucleotide state (S2 Table). Notably, the signature motif is located at the N-terminal end of h5 helix. As the signature motif retracts from the ADP molecule and opens the active site (S1B and S1C Fig), the retraction motion could be directly transmitted along the helix to the C-terminal end, and be delivered through the hydrogen bond involving Gln143 to the L-loop (Fig 4A), leading to the separation of two L-loops.

For the coupling motions at the cytoplasmic end of TMD, we compared the snapshots having the widest opening cytoplasmic gate II with the crystal structure of the occluded state, and found that opening of the cytoplasmic gate not only leads to separation of two exTM3 stretches, but is also accompanied by the outward movements of the cytoplasmic ends of TM2, TM6 and TM7 helices (Fig 4B and S5 Fig), indicating that the cytoplasmic ends of these segments would move cooperatively with the cytoplasmic gate II. Further inspection at the region corroborates this notion by revealing extensive hydrophobic interactions between the cytoplasmic ends of TM2, TM6 and TM7 helices as well as the L-loop, which involve the contributions from Ile72, Val76 and Leu80 on TM2, Ile204 on TM6, Leu226, Leu228 and Leu235 on TM7, and Met211, Met213 and Leu214 on the L-loop (Fig 4C). These interactions indicate that the TM2/6/7 helices could be largely viewed as a helix bundle which would move collectively with the L-loop. The collective motion is further confirmed by the linear correlation between d_L-loop_ and the distance between the cytoplasmic ends of two TM2 helices represented by Gln78 (d_Q78_) (Fig 4F). Intriguingly, equivalent hydrophobic interaction network could also be found in other two type II importers with high-resolution structures: the molybdate/tungstate transporter MolBC (Fig 4D) and the *Yersinia pestis* heme transporter HmuUV (Fig 4E), indicating a common coupling mechanism adopted by the type II importers.

Besides the hydrophobic interactions coupling the L-loop and the TM2/6/7 helix bundle, we found that the helix bundle is also coupled with cytoplasmic gate II through hydrogen bond interactions involving a conserved Asn83 lying at the cytoplasmic end of exTM3 stretch [17]. The asparagine residue, on one hand, forms two hydrogen bonds with Gln78 and Phe81 (occurrence = ~35% and ~24%, respectively, S3 Table), which lie at the cytoplasmic end of TM2 helix (Fig 4C). These two hydrogen bonds plus the covalent backbone connecting Asn83 and TM2 helix couple the motions of TM2 helix to those of Asn83, as shown by the linear correlation (Fig 4G) between d_Q78_ and the distance between two Asn83 (d_N83_). On the other hand, Asn83 also forms two strong hydrogen bonds with the backbone nitrogen atoms of Leu85 and Ala86 on exTM3 stretch using its side chain and backbone carbonyl oxygen, respectively (Fig 4C and S3 Table), through which it is tightly coupled with the cytoplasmic gate II as shown by the linear correlation between d_L85_ and d_N83_ (Fig 4H). Notably, similar hydrogen bond network was also found in MolBC (Fig 4D) and HmuUV (Fig 4E). Thus, the hydrophobic interactions inside the TM2/6/7 helix bundle plus the L-loop and the Asn83-involved hydrogen bond interactions constitute a contiguous interaction network, which transmits the separating motion of L-loops to the cytoplasmic gate II.

It should be noted that the coupling motions between active site and L-loops, and between L-loops and cytoplasmic gate II are not extremely tight. In other words, opening of the ADP-bound site does not necessarily lead to the separation of L-loops (Figs 1D, 1E, 3A and 3B, see **ATP/ADP.IP** trajectory 2 and **ADP.IP/ATP** trajectory 5 for example), and the separation of L-loops does not necessarily lead to cytoplasmic gate II opening (Fig 3B and 3F, see the last 40 ns of **ADP.IP/ATP** trajectory 2 for example). Moreover, it should also be noted that the contiguous interaction network plus the TMD-NBD interface deduced from **ATP/ADP.IP** trajectory 5 and **ADP.IP/ATP** trajectory 2 may constitute only one of the possible pathways coupling the active site and cytoplasmic gate II. Other pathways are still possible as implicated by other trajectories of our simulation. One example is **ATP/ADP.IP** trajectory 1, which shows visibly long-lasting opening of cytoplasmic gate II (Fig 3E) without evident changes in the ADP-bound site (Fig 1D and S1B Fig) and the L-loops (Fig 3A).

## Discussion

Although the arrangement of the closed NBD dimer interface with ATP bound has been confirmed by various experimental and computational studies for years, the exact conformational changes upon ATP hydrolysis are still under debate. A number of models have been proposed for the process and they can be divided into two types according to the level of separation between two NBDs after hydrolysis [56]. In the association/dissociation model, two NBDs lose contact after ATP hydrolysis and the WA and the juxtaposing signature motifs of both sites move far away from each other. This type of models involve the tweezers-like [57], ABC switch [58, 59], and processive clamp [60] models *etc*., which differ in the description of the number of hydrolyzed ATPs and the response of each nucleotide-binding site to the variation of nucleotide state. In the constant-contact model, however, the NBDs never lose contact during the hydrolysis cycle and only one site would open to exchange hydrolysis products for a new ATP [33].

In this work, we monitor the conformational variation of the type II ABC importer BtuCD-F upon hydrolysis of one ATP molecule using MD simulations. 1.5 μs trajectories were produced for the **ATP/ADP.IP** (ATP hydrolyzes at site 2) and the **ADP.IP/ATP** (ATP hydrolyzes at site 1) systems, respectively. We found that for each system, four out of five parallel trajectories showed obvious asymmetric opening of the nucleotide binding sites. H-bonds between nucleotide and signature motif at the ADP-bound site were evidently disrupted, whereas those at the ATP-bound site were mostly retained (Fig 1C–1F). These simulation results seem to be supportive to the constant-contact model, at least in the early stage after single ATP hydrolysis. However, it is worth noting that the conventional MD simulation method employed in this study limits the sampling time to sub-microsecond scale and the wide separation of NBDs may take place in much longer time scale. Therefore, based on the current simulations we can only infer that NBD dimer tends to open asymmetrically at the early stage of ATP hydrolysis. Because the constant-contact model entails more limited variations at the NBD dimer interface, this model may be questioned as how ADP exits from the site and how TMDs response to the relatively small motions in NBDs. However, our simulations show that opening of the active site could leave enough space for the exit of ADP and IP (Fig 2B), indicating that nucleotide exchange could be achieved even if only one active site is opened. Disengagement of the adenine moiety from the conserved A-loop was also observed, which could be regarded as the initial step of ADP exit [33]. Besides the changes in NBDs, the simulations also show evident separation of the L-loops and long-lasting opening of the cytoplasmic gate II when only one active site was opened (Fig 3). These observations indicate that the constant-contact model is effective in triggering changes at the translocation pathway and probably also in facilitating substrate transport. This notion is consistent with the experimental observation that the BtuCD mutant with one of the ATPase sites abolished still retains an appreciable portion of ATPase activity and transport capability that support full utilization of B_12_ *in vivo* [61].

Though our simulations mainly support the constant-contact type model, they do not rule out the association/dissociation type models. In the last 250 ns of **ATP/ADP.IP** trajectory 1, last 45 ns of **ATP/ADP.IP** trajectory 5 (Fig 1C) and the last 70 ns of **ADP.IP/ATP** trajectory 4 (Fig 1F), disruption of more than half of the H-bonds at the ATP-bound site was observed, indicating that both sites may disrupt after ATP hydrolysis, leading to complete dissociation of the NBD interface. Disruption of both ATP-bound and ADP-bound sites has also been observed in the MalK dimer [36], and the changes are more dramatic in that case (both active sites separated by about 4 Å). Interestingly, a recent luminescence resonance energy transfer (LRET) study on an ABC exporter MsbA [62] reported that the constant-contact mode is predominant at lower temperature (30°C), but the NBD dissociation rate is obviously increased supporting the association/dissociation type models at higher temperature (37°C). The LRET experiment indicates that the association/dissociation and constant-contact models are not mutually exclusive. Both types of models can be adopted by the same transporter and the transporter may follow either type of models under certain conditions. Thus, the real question for the post-hydrolysis process could be which of the two models dominates or which model is more efficient in propelling substrate transport.

The L-loop has been proposed to play an important role in coupling the motion of NBDs to the conformational changes of the cytoplasmic gate since the first high-resolution BtuCD crystal structure was obtained [19]. Crystal structures solved at various nucleotide/BtuF bound states subsequently support this notion through structural comparison [17, 18, 20]. Our simulations, however, provide direct evidence of the coupling motions between NBDs and TMDs upon ATP hydrolysis through L-loop. The TMD-NBD interface formed between the L-loop and the h5 helix of NBD (Fig 4A and S2 Table), the hydrophobic interactions at the cytoplasmic end of TM2/6/7 helix bundle plus the L-loop, and the Asn83-involved hydrogen bond interactions connecting TM2 helix to the cytoplasmic gate II (Fig 4C and S3 Table) could be responsible for the coupling motions. Similar interactions can be found in another two type II importers: the *Haemophilus influenzae* molybdate/tungstate transporter MolBC (Fig 4D) and the *Yersinia pestis* heme transporter HmuUV (Fig 4E), indicating that the coupling mechanism could be a general feature for all type II importers.

**Fig 5:**
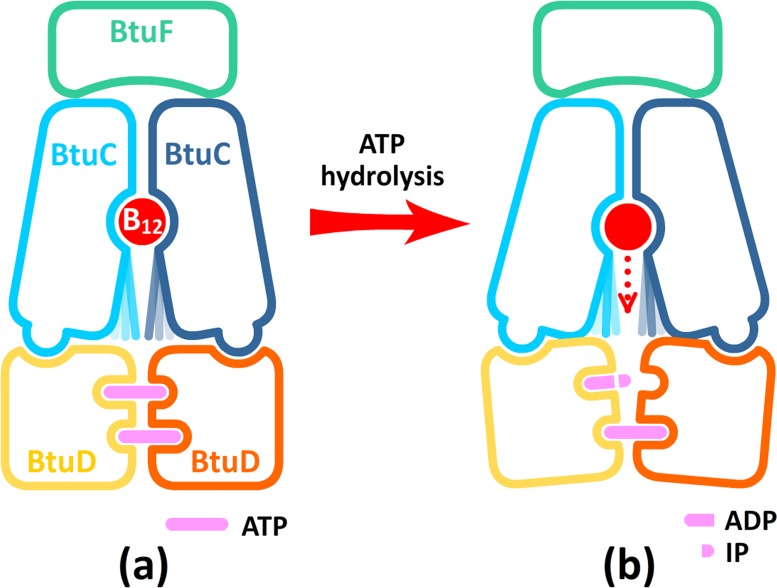
Schematic diagram of the conformational changes triggered by hydrolysis of one ATP at the active site. The swinging exTM3 stretches are represented by blue or cyan straight lines, and the L-loops are represented by the knobs on BtuC subunits.

Based on these observations, a plausible mechanism of ATP hydrolysis-driven conformational conversion can be proposed for type II ABC importers such as BtuCD (Fig 5). Upon BtuF and nucleotide binding, the importer turns into the occluded state and accommodates B_12_ molecule in the middle of the translocation pathway. Swinging motion of the exTM3 stretches (which constitute the cytoplasmic gate II) could be observed [23], but the cytoplasmic gate II is still impermeable for the large-sized substrate (Fig 5A). After hydrolysis of one of the binding ATPs, one active site is disrupted (Fig 5B). Though two NBDs still keep in contact mainly through the ATP-bound site, they move away from each other and drive separation of the L-loops through the TMD-NBD interface. Motions of the L-loops are transmitted through the contiguous interaction network involving Asn83 and TM2/6/7 helix bundle to the cytoplasmic gate II, leading to long-lasting opening of gate II, which effectively raises the probability of B_12_ translocation (Fig 5B).

Though the widely opened state of cytoplasmic gate II was observed in our simulation, which is very close to the inward-facing conformation of the molybdate/tungstate transporter MolBC [63], permeation of the large-size substrate has not been observed possibly due to the limited simulation time (S4 Fig). Enhanced sampling methods and free energy calculations may be utilized to circumvent the sampling problem. These methods may delineate the details of B_12_ translocation through the cytoplasmic gates and shed light on the physical nature of the driving force propelling the translocation process [19].

Lastly, it should be noted that we simulated ATP hydrolysis through a conventional method by directly replacing ATP with its hydrolysis products and initiating MD simulation using Maxwell velocity distribution. This method assumes that the chemical energy released from the terminal phosphoanhydridic bond of ATP quickly dissipates to the environment and does not alter the dynamics of transporter. However, recent single-molecule fluorescence correlation spectroscopy data indicate that the energy released by chemical reaction could generate an asymmetric pressure wave and result in an enhanced diffusion of a single enzyme molecule [64]. As ABC transporter could also be viewed as an enzyme and ATP hydrolysis releases considerable energy of 30.5 kJ/mol, the produced pressure wave is very likely to disturb the dynamics of nucleotide binding site (by hastening the opening of active site, maybe) as well as the rest part of transporter. Thus, new theories and methods are needed to take the released energy into account and to provide a more realistic description for the conformational changes triggered by ATP hydrolysis.

## Supporting Information

S1 Fig (a-b) Variations of the distances between the mass center of alpha and beta phosphate groups of nucleotide and the mass center of the corresponding signature motif ^128^SGGE^131^ (d_nuc-sig_) at two active sites of the **ATP/ADP.IP** system along simulation time. (c-d) Variations of d_nuc-sig_ at two active sites of the **ADP.IP/ATP** system. (e-f) Variations of d_nuc-sig_ at two active sites of the **ATP/ATP** system obtained from our previous work. The values of d_nuc-sig_ in the crystal structures of the occluded state (occ-BtuCD, PDBID: 4FI3) and the AMP-PNP-bound BtuCD (out^II^-BtuCD, PDBID: 4R9U) are marked by yellow and cyan horizontal lines, respectively.
(TIF)

S2 Fig Variations of d_Mg-βP_ (a) and d_Mg-γP_ (b) at site 1 and variations of d_Mg-βP’_ (c) and d_Mg-IP_ (d) at site 2 of the **ATP/ADP.IP** system along simulation time. d_Mg-βP_ and d_Mg-γP_ denote the distance between magnesium ion and the βP or γP atom of ATP in the ATP-bound site, respectively. d_Mg-IP_ denotes the distance between magnesium ion and the phosphorus atom of IP at the ADP-bound site. d_Mg-βP’_ denotes the distance between magnesium ion and the βP atom of ADP. Variations of d_Mg-βP’_ (e) and d_Mg-IP_ (f) at site 1 and variations of d_Mg-βP_ (g) and d_Mg-γP_ (h) at site 2 of the **ADP.IP/ATP** system. Variations of d_Mg-βP_ (i) and d_Mg-γP_ (j) at site 1 and those at site 2 (k and l) of the **ATP/ATP** system.
(TIF)

S3 Fig Projections of the trajectories of the **ATP/ADP.IP** (a), the **ADP.IP/ATP** (b), and the **ATP/ATP** (c) systems on the 2-dimensional space spanned by d_S143_ and d_L85_. d_S143_ is defined as the distance between C_α_ atoms of Ser143 on the cytoplasmic ends of TM5 helices. The projections of the crystal structures of the occluded state (occ-BtuCD, PDBID: 4FI3), the nucleotide-free BtuCD (out^I^-BtuCD, PDBID: 1L7V), the AMP-PNP-bound BtuCD (out^II^-BtuCD, PDBID: 4R9U) and the asymmetrical nucleotide-free BtuCD-F complex (asym-BtuCD, PDBID: 2QI9), and the homological model of inward-facing state of BtuCD using MolBC as template (in-BtuCD) are also plotted, respectively. Data of the **ATP/ATP** system were obtained from our previous work.
(TIF)

S4 Fig Variations of d_B12-M176s_ (the distance between the cobalt atom of B_12_ and the center of two C_α_ atoms of Met176 residues on TM5 helices) in five 300-ns trajectories of the **ATP/ADP.IP** (a), the **ADP.IP/ATP** (b) and the **ATP/ATP** (c) systems along simulation time. d_B12-M176s_ is defined as the distance between Co^3+^ of the substrate and the center of C_α_ atoms of Met176 on TM5a helices which form the periplasmic gate. Data of the **ATP/ATP** system were obtained from our previous work.
(TIF)

S5 Fig Snapshots of the cytoplasmic ends of TMDs with the largest values of d_L85_ viewed from the periplasm. (a) 126.90 ns of **ADP.IP/ATP** trajectory 2 (d_L85_ = 13.6 Å). (b) 5.55 ns of **ATP/ADP.IP** trajectory 5 (d_L85_ = 13.8 Å). (c) 68.20 ns at **ATP/ADP.IP** trajectory 5 (d_L85_ = 13.4 Å). The importer is represented by ribbons. The segments are colored by cyan and blue for two TMDs, respectively, and TM2, exTM3, TM6 and TM7 are highlighted by pink and purple for two TMDs, respectively. Leu85 and Asn83 on exTM3 stretch are represented as balls. The segments in the crystal structure are colored yellow. The L-loops are omitted for clarity.
(TIF)

S1 Table Occurrences of the hydrogen bond interactions between the WA motif and the binding nucleotide. ^a^ Hydrogen bond is defined as the donor and acceptor atoms are within 3.5 Å from each other, and the angle formed by the donor, hydrogen, and acceptor atoms is less than 60° from 180°.
(PDF)

S2 Table Occurrences of the hydrogen bond interactions across the TMD-NBD interface in the 300-ns trajectories. ^a^ Hydrogen bond is defined the same as described in S1 Table. Only the hydrogen bond interactions with occurrence of over 30% are listed. ^b^ Average occurrence of hydrogen bond in five parallel 300-ns trajectories of the system. ^c^ Standard deviation of the occurrences of hydrogen bond in five parallel trajectories.
(PDF)

S3 Table Occurrences of the Asn83-involved hydrogen bond interactions. ^a^ Hydrogen bond is defined the same as described in S1 Table. ^b^ Average occurrence of hydrogen bond in five parallel 300-ns trajectories of the system. ^c^ Standard deviation of the occurrence of hydrogen bond in five parallel trajectories.
(PDF)
