# Supplementary figures and images for: ATP Hydrolysis Induced Conformational Changes in the Vitamin B12 Transporter BtuCD Revealed by MD Simulations
Source: PLoS One. 2016 Nov 21;11(11):e0166980. doi: 10.1371/journal.pone.0166980 (PMC5117765; doi:10.1371/journal.pone.0166980)

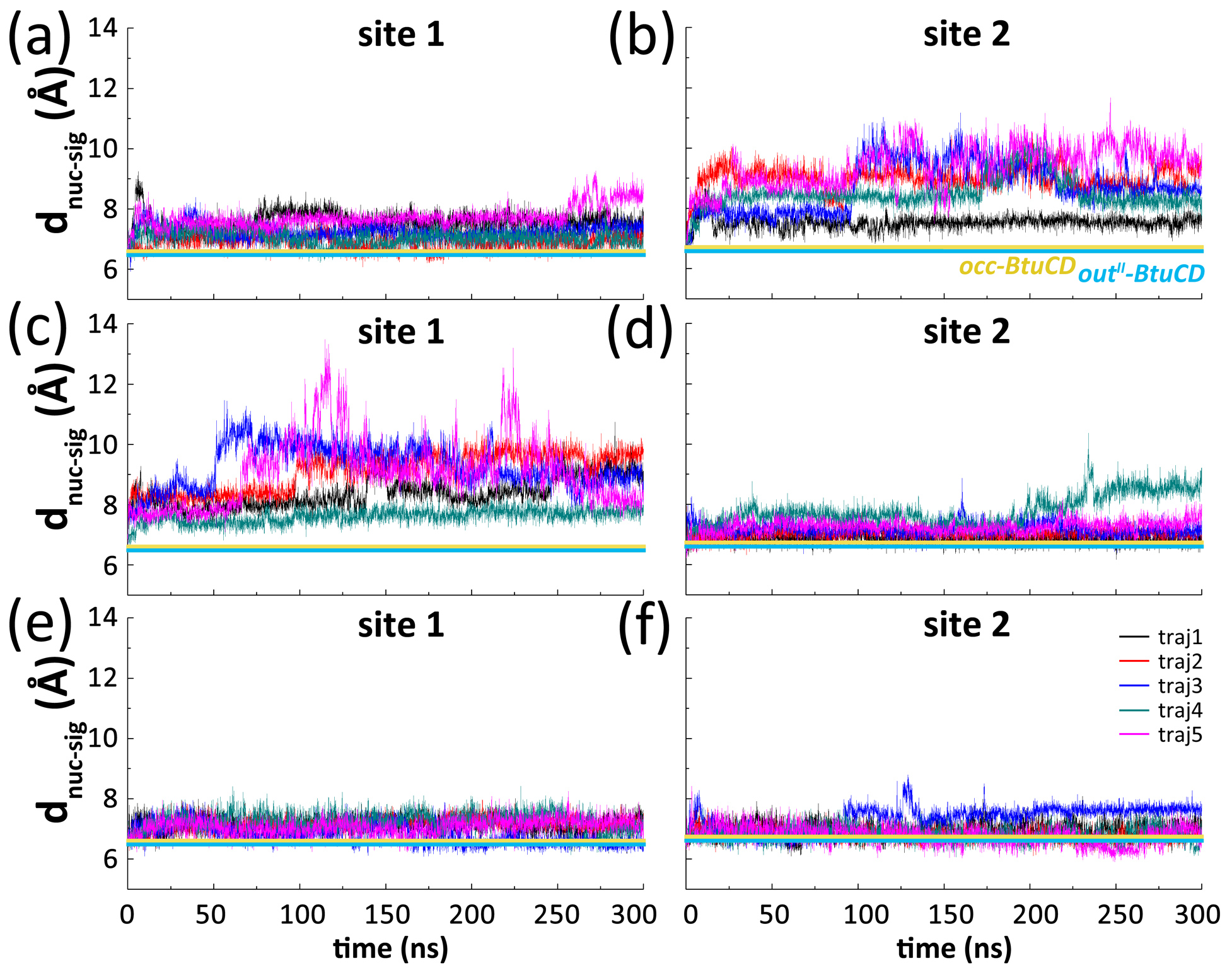

Supplement: S1 Fig — (a-b) Variations of the distances between the mass center of alpha and beta phosphate groups of nucleotide and the mass center of the corresponding signature motif 128SGGE131 (dnuc-sig) at two active sites of the ATP/ADP.IP system along simulation time. (c-d) Variations of dnuc-sig at two active sites of the ADP.IP/ATP system. (e-f) Variations of dnuc-sig at two active sites of the ATP/ATP system obtained from our previous work. The values of dnuc-sig in the crystal structures of the occluded state (occ-BtuCD, PDBID: 4FI3) and the AMP-PNP-bound BtuCD (outII-BtuCD, PDBID: 4R9U) are marked by yellow and cyan horizontal lines, respectively. (TIF) [file pone.0166980.s001.tif]

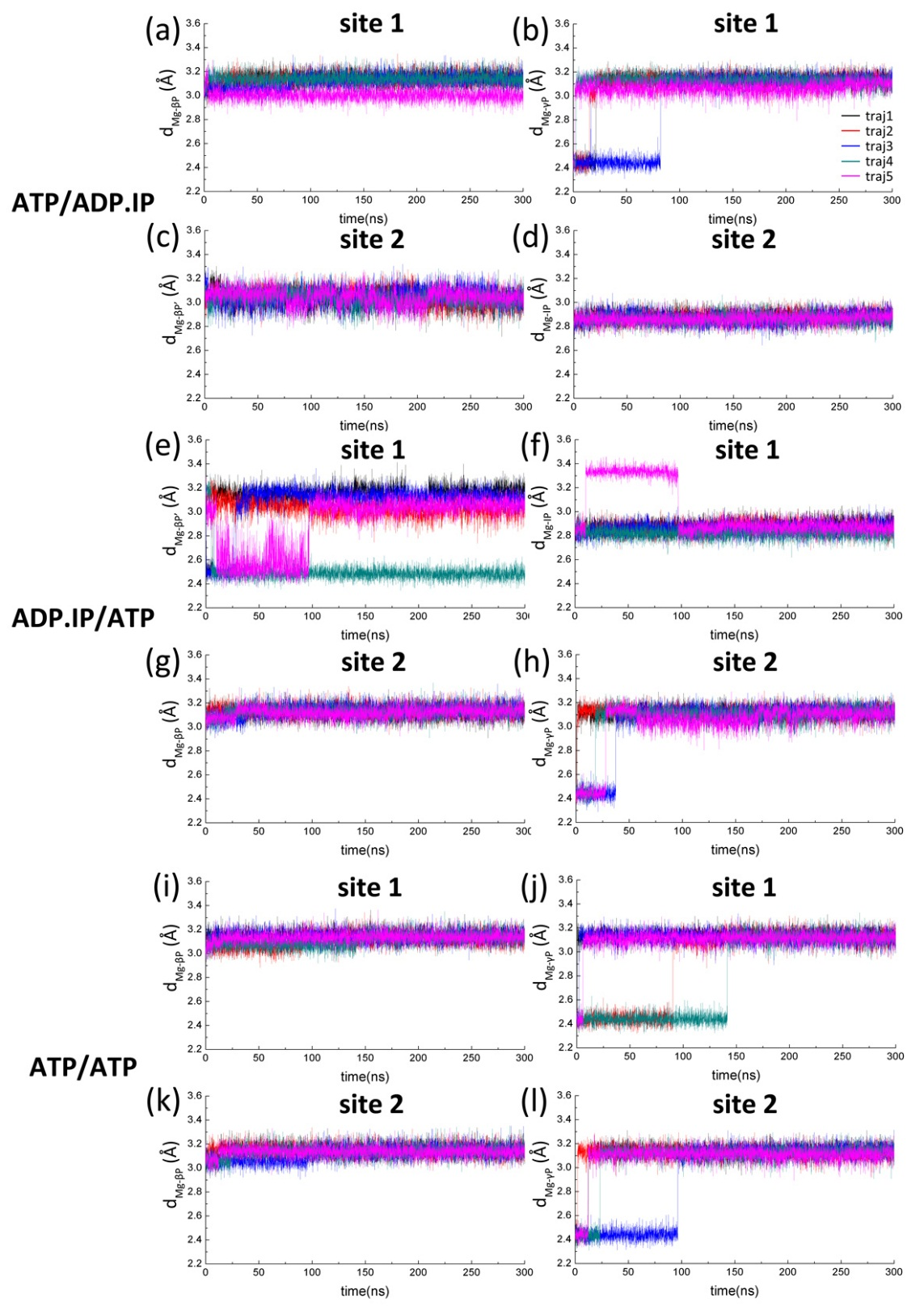

Supplement: S2 Fig — Variations of dMg-βP (a) and dMg-γP (b) at site 1 and variations of dMg-βP’ (c) and dMg-IP (d) at site 2 of the ATP/ADP.IP system along simulation time. dMg-βP and dMg-γP denote the distance between magnesium ion and the βP or γP atom of ATP in the ATP-bound site, respectively. dMg-IP denotes the distance between magnesium ion and the phosphorus atom of IP at the ADP-bound site. dMg-βP’ denotes the distance between magnesium ion and the βP atom of ADP. Variations of dMg-βP’ (e) and dMg-IP (f) at site 1 and variations of dMg-βP (g) and dMg-γP (h) at site 2 of the ADP.IP/ATP system. Variations of dMg-βP (i) and dMg-γP (j) at site 1 and those at site 2 (k and l) of the ATP/ATP system. (TIF) [file pone.0166980.s002.tif]

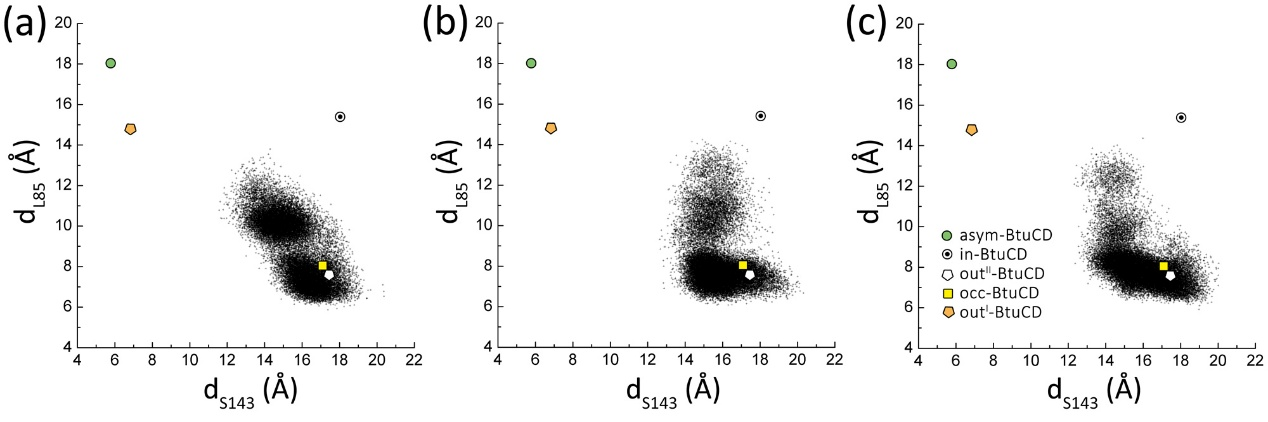

Supplement: S3 Fig — Projections of the trajectories of the ATP/ADP.IP (a), the ADP.IP/ATP (b), and the ATP/ATP (c) systems on the 2-dimensional space spanned by dS143 and dL85. dS143 is defined as the distance between Cα atoms of Ser143 on the cytoplasmic ends of TM5 helices. The projections of the crystal structures of the occluded state (occ-BtuCD, PDBID: 4FI3), the nucleotide-free BtuCD (outI-BtuCD, PDBID: 1L7V), the AMP-PNP-bound BtuCD (outII-BtuCD, PDBID: 4R9U) and the asymmetrical nucleotide-free BtuCD-F complex (asym-BtuCD, PDBID: 2QI9), and the homological model of inward-facing state of BtuCD using MolBC as template (in-BtuCD) are also plotted, respectively. Data of the ATP/ATP system were obtained from our previous work. (TIF) [file pone.0166980.s003.tif]

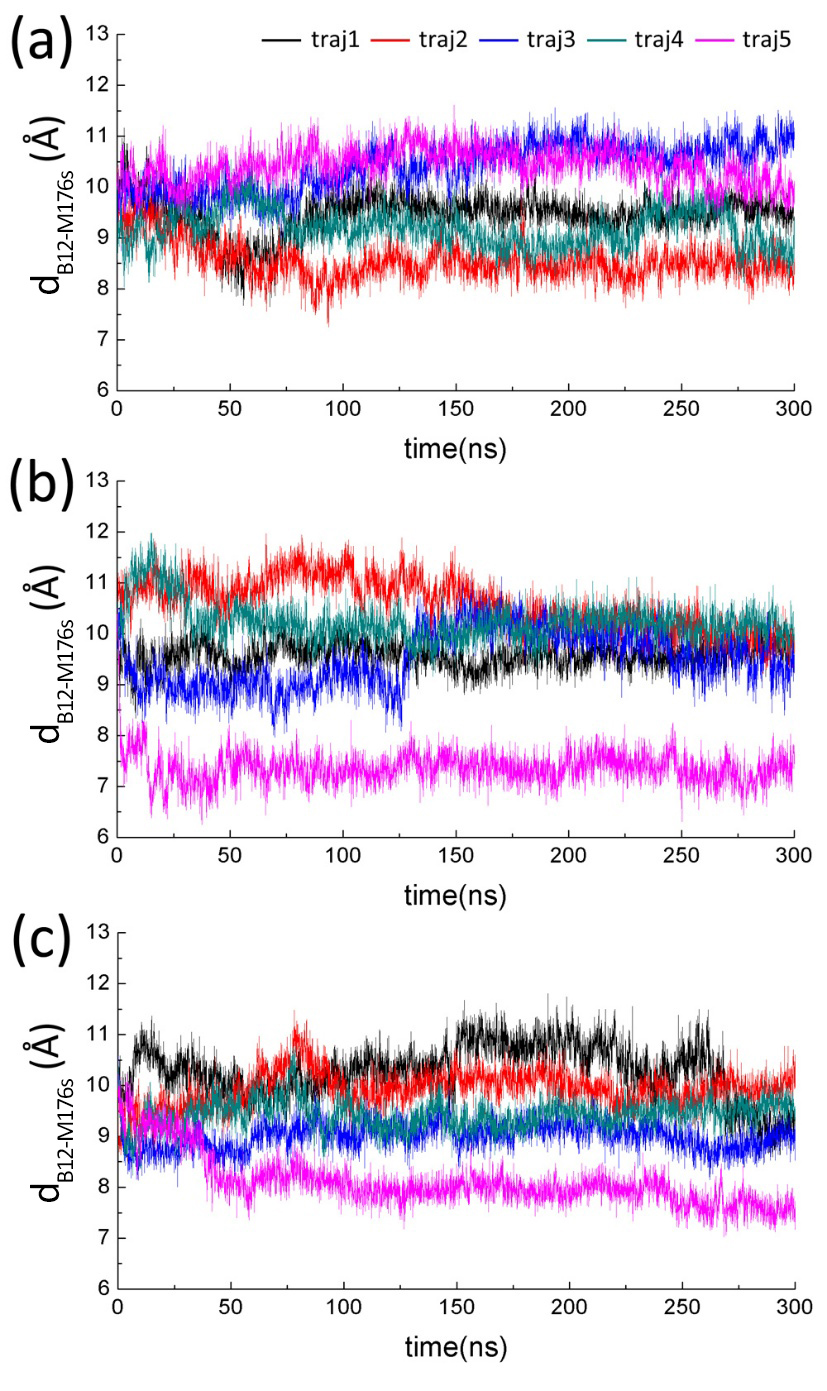

Supplement: S4 Fig — Variations of dB12-M176s (the distance between the cobalt atom of B12 and the center of two Cα atoms of Met176 residues on TM5 helices) in five 300-ns trajectories of the ATP/ADP.IP (a), the ADP.IP/ATP (b) and the ATP/ATP (c) systems along simulation time. dB12-M176s is defined as the distance between Co3+ of the substrate and the center of Cα atoms of Met176 on TM5a helices which form the periplasmic gate. Data of the ATP/ATP system were obtained from our previous work. (TIF) [file pone.0166980.s004.tif]

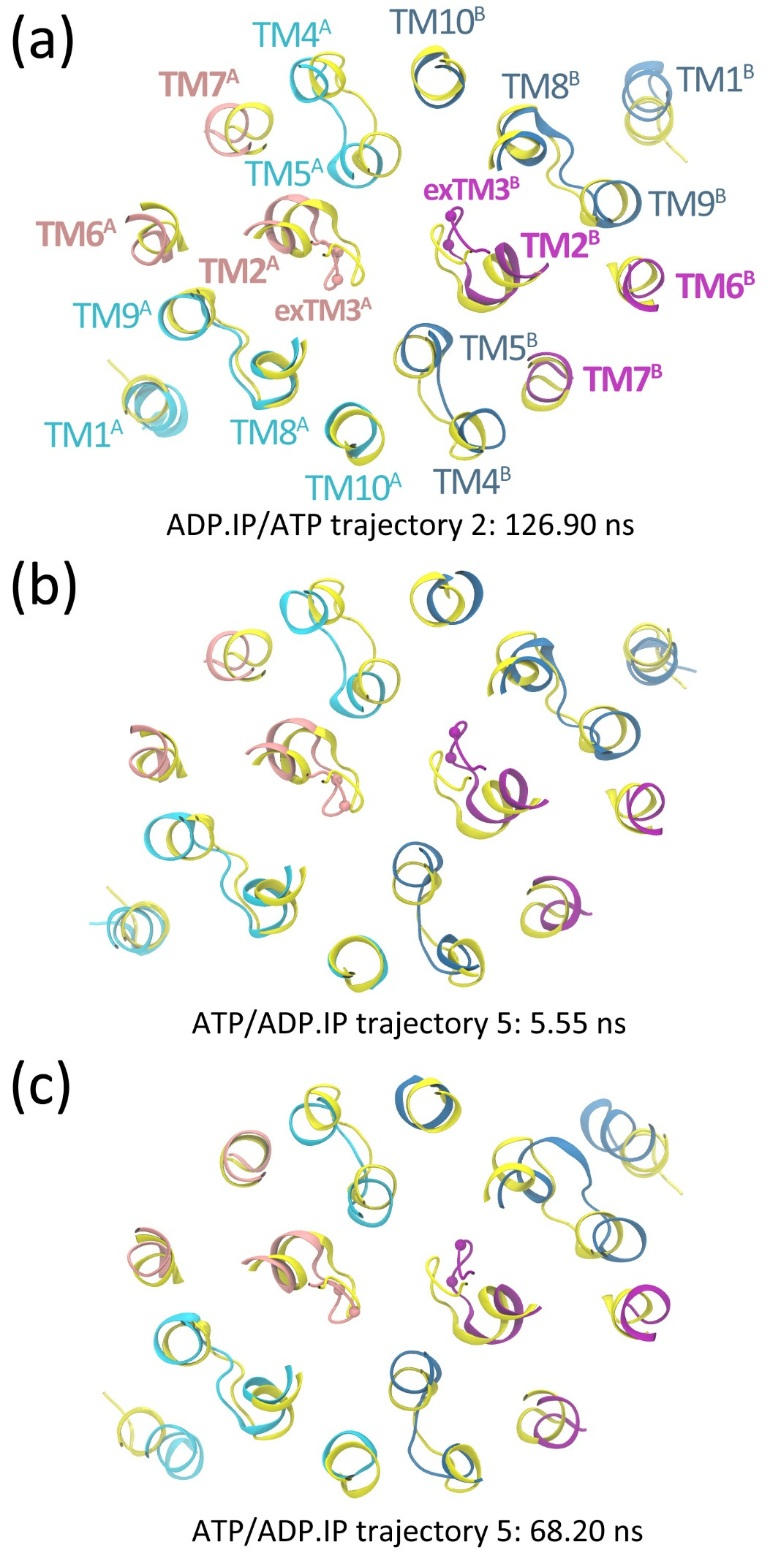

Supplement: S5 Fig — (a) 126.90 ns of ADP.IP/ATP trajectory 2 (dL85 = 13.6 Å). (b) 5.55 ns of ATP/ADP.IP trajectory 5 (dL85 = 13.8 Å). (c) 68.20 ns at ATP/ADP.IP trajectory 5 (dL85 = 13.4 Å). The importer is represented by ribbons. The segments are colored by cyan and blue for two TMDs, respectively, and TM2, exTM3, TM6 and TM7 are highlighted by pink and purple for two TMDs, respectively. Leu85 and Asn83 on exTM3 stretch are represented as balls. The segments in the crystal structure are colored yellow. The L-loops are omitted for clarity. (TIF) [file pone.0166980.s005.tif]
