# Supplementary material for: ATP Hydrolysis Induced Conformational Changes in the Vitamin B12 Transporter BtuCD Revealed by MD Simulations
Source: PLoS One. 2016 Nov 21;11(11):e0166980. doi: 10.1371/journal.pone.0166980 (PMC5117765; doi:10.1371/journal.pone.0166980)
Supplement: S1 Table — a Hydrogen bond is defined as the donor and acceptor atoms are within 3.5 Å from each other, and the angle formed by the donor, hydrogen, and acceptor atoms is less than 60° from 180°. (PDF) [file pone.0166980.s006.pdf]

S1 Table. Occurrences of the hydrogen bond interactions between the WA motif and the binding nucleotide.

site 1	occurrence (%)[a]														
	ATP/ADP.IP					ADP.IP/ATP					ATP/ATP				
**residue**	**1**	**2**	**3**	**4**	**5**	**1**	**2**	**3**	**4**	**5**	**1**	**2**	**3**	**4**	**5**
Asn35	99	95	99	100	100	0	7	0	0	11	100	95	99	97	95
Gly36	100	100	98	100	100	99	100	99	100	99	99	98	100	100	100
Ala37	32	25	29	25	42	81	59	62	73	69	26	36	20	26	38
Gly38	95	92	91	94	99	96	98	32	88	98	88	89	90	94	89
Lys39	100	100	100	100	100	100	100	99	100	100	100	100	100	100	100
Ser40	100	100	100	100	100	100	80	99	94	82	100	100	100	100	100
Thr41	100	100	100	100	100	100	100	10	100	100	100	100	100	100	100
**site 2**															
Asn35	2	61	6	33	37	0	88	100	88	95	100	99	98	100	99
Gly36	99	100	100	100	100	99	100	98	99	100	99	99	99	100	100
Ala37	87	60	50	56	45	21	22	22	33	29	27	21	30	26	32
Gly38	96	97	99	99	98	98	95	95	97	95	95	91	93	95	94
Lys39	100	100	100	100	100	100	100	100	100	100	100	100	100	100	100
Ser40	44	56	72	73	69	100	100	100	100	100	100	100	100	100	100
Thr41	100	100	100	100	100	100	100	100	100	100	100	100	100	100	100

[a] Hydrogen bond is defined as the donor and acceptor atoms are within 3.5 Å from each other, and the angle formed by the donor, hydrogen, and acceptor atoms is less than 60° from 180°.
