# Supplementary material for: ATP Hydrolysis Induced Conformational Changes in the Vitamin B12 Transporter BtuCD Revealed by MD Simulations
Source: PLoS One. 2016 Nov 21;11(11):e0166980. doi: 10.1371/journal.pone.0166980 (PMC5117765; doi:10.1371/journal.pone.0166980)
Supplement: S2 Table — a Hydrogen bond is defined the same as described in S1 Table. Only the hydrogen bond interactions with occurrence of over 30% are listed. b Average occurrence of hydrogen bond in five parallel 300-ns trajectories of the system. c Standard deviation of the occurrences of hydrogen bond in five parallel trajectories. (PDF) [file pone.0166980.s007.pdf]

S2 Table. Occurrences of the hydrogen bond interactions across the TMD-NBD interface in the 300-ns trajectories.

TMD	NBD	occurrence (%)[a]					
chain A	chain C	ATP/ADP.IP		ADP.IP/ATP		ATP/ATP	
		**avg**[b]	stdev[c]	**avg**	stdev	**avg**	stdev
Gln8	Trp91	**48.0**	29.4	**59.0**	34.0	**34.3**	35.0
Gln8	Gly121	**36.5**	23.1	**41.1**	34.2	**22.6**	27.1
Arg209	Leu96	**40.4**	13.7	**29.7**	14.7	**26.7**	13.0
Ser220	Ser79	**2.1**	2.6	**33.4**	35.3	**4.2**	8.6
Arg222	Met49	**49.1**	8.8	**48.4**	15.6	**57.1**	8.8
Gln223	Arg75	**90.9**	4.3	**82.5**	11.4	**89.1**	4.0
Gln223	Tyr77	**92.1**	1.9	**92.9**	2.5	**89.4**	3.2
Gln223	Gln143	**100.0**	0.0	**100.0**	0.0	**100.0**	0.0
Thr270	Ala87	**36.3**	18.4	**29.5**	21.4	**40.1**	19.4
**chain B**	**chain D**	**occurrence (%)**					
Glu1	Glu105	**5.6**	3.7	**8.4**	4.1	**34.1**	33.5
Leu2	Thr104	**12.2**	15.8	**24.6**	12.6	**41.4**	34.4
Gln8	Trp91	**50.5**	42.3	**50.1**	43.7	**49.4**	44.4
Gln8	Gly121	**36.7**	35.5	**39.2**	37.0	**33.7**	31.5
Arg209	Leu96	**37.5**	8.4	**32.4**	15.7	**28.1**	13.0
Asn212	His92	**8.5**	11.6	**24.7**	37.4	**46.0**	29.7
Ser220	Gln82	**16.1**	4.8	**27.7**	24.1	**36.4**	11.7
Arg222	Met49	**48.2**	14.0	**45.4**	14.8	**58.9**	14.9
Gln223	Arg75	**87.6**	2.5	**85.1**	5.4	**84.6**	4.1
Gln223	Tyr77	**92.3**	0.9	**93.4**	1.6	**91.2**	3.0
Gln223	Gln143	**100.0**	0.0	**100.0**	0.0	**100.0**	0.0
Thr270	Ala87	**41.2**	9.1	**33.4**	18.3	**13.4**	22.2

[a] Hydrogen bond is defined the same as described in S1 Table. Only the hydrogen bond interactions with occurrence of over 30% are listed.

[b] Average occurrence of hydrogen bond in five parallel 300-ns trajectories of the system.

[c] Standard deviation of the occurrences of hydrogen bond in five parallel trajectories.
