# Supplementary material for: ATP Hydrolysis Induced Conformational Changes in the Vitamin B12 Transporter BtuCD Revealed by MD Simulations
Source: PLoS One. 2016 Nov 21;11(11):e0166980. doi: 10.1371/journal.pone.0166980 (PMC5117765; doi:10.1371/journal.pone.0166980)
Supplement: S3 Table — a Hydrogen bond is defined the same as described in S1 Table. b Average occurrence of hydrogen bond in five parallel 300-ns trajectories of the system. c Standard deviation of the occurrence of hydrogen bond in five parallel trajectories. (PDF) [file pone.0166980.s008.pdf]

S3 Table. Occurrences of the Asn83-involved hydrogen bond interactions.

hydrogen bond		occurrence (%)[a]					
TMD[A]		ATP/ADP.IP		ADP.IP/ATP		ATP/ATP	
		**avg**[b]	stdev[c]	**avg**	stdev	**avg**	stdev
Asn83	Gln78	**30.9**	8.1	**42.7**	7.5	**34.7**	8.9
	Phe81	**25.0**	13.5	**16.1**	7.6	**22.6**	9.0
	Leu85	**98.2**	0.7	**97.7**	0.9	**97.6**	1.2
	Ala86	**84.6**	4.2	**76.7**	9.1	**85.5**	8.7
**TMD[B]**		**occurrence (%)**					
Asn83	Gln78	**38.9**	18.1	**29.9**	8.5	**30.9**	11.7
	Phe81	**19.6**	7.4	**28.2**	8.0	**28.5**	6.1
	Leu85	**96.8**	2.3	**98.5**	1.3	**97.7**	1.8
	Ala86	**86.4**	3.7	**85.4**	5.9	**84.9**	2.4

[a] Hydrogen bond is defined the same as described in S1 Table.

[b] Average occurrence of hydrogen bond in five parallel 300-ns trajectories of the system.

[c] Standard deviation of the occurrence of hydrogen bond in five parallel trajectories.
